# Widespread occurrence of *N*^6^-methyladenosine in bacterial mRNA

**DOI:** 10.1093/nar/gkv596

**Published:** 2015-06-11

**Authors:** Xin Deng, Kai Chen, Guan-Zheng Luo, Xiaocheng Weng, Quanjiang Ji, Tianhong Zhou, Chuan He

**Affiliations:** 1 TEDA Institute of Biological Sciences and Biotechnology, Nankai University, 23 Hongda Street, Tianjin 300457, P.R. China; 2 Key Laboratory of Molecular Microbiology and Technology, Ministry of Education, Tianjin 300071, P.R. China; 3 Department of Chemistry and Institute for Biophysical Dynamics, The University of Chicago, 929 East 57th Street, Chicago, Illinois 60637, USA; 4 Howard Hughes Medical Institute, The University of Chicago, 929 East 57th Street, Chicago, Illinois 60637, USA

## Abstract

*N*^6^-methyladenosine (m^6^A) is the most abundant internal modification in eukaryotic messenger RNA (mRNA). Recent discoveries of demethylases and specific binding proteins of m^6^A as well as m^6^A methylomes obtained in mammals, yeast and plants have revealed regulatory functions of this RNA modification. Although m^6^A is present in the ribosomal RNA of bacteria, its occurrence in mRNA still remains elusive. Here, we have employed ultra-high pressure liquid chromatography coupled with triple-quadrupole tandem mass spectrometry (UHPLC-QQQ-MS/MS) to calculate the m^6^A/A ratio in mRNA from a wide range of bacterial species, which demonstrates that m^6^A is an abundant mRNA modification in tested bacteria. Subsequent transcriptome-wide m^6^A profiling in *Escherichia coli* and *Pseudomonas aeruginosa* revealed a conserved m^6^A pattern that is distinct from those in eukaryotes. Most m^6^A peaks are located inside open reading frames and carry a unique consensus motif of GCCAU. Functional enrichment analysis of bacterial m^6^A peaks indicates that the majority of m^6^A-modified genes are associated with respiration, amino acids metabolism, stress response and small RNAs, suggesting potential functional roles of m^6^A in these pathways.

## INTRODUCTION

*N*^6^-methyladenosine (m^6^A) is the most frequent internal mRNA modification in a wide range of eukaryotes and certain viral RNAs (1–6). Modification of m^6^A is mediated by an *N*^6^-adenosine methyltransferase complex including a 70-kD SAM (S-adenosylmethionine)-binding subunit methyltransferase like 3 (METTL3, also called MT-A70), methyltransferase like 14 (METTL14) and Wilms tumor 1 associated protein (WTAP) (7). METTL3 and METTL14 form a heterodimer that catalyzes m^6^A RNA methylation, while WTAP interacts with the complex and affects the mRNA methylation (8). These methyltransferases play important roles in mouse embryonic stem cell differentiation and circadian rhythms (9–11) Fat mass and obesity-associated protein (FTO) and *alkB* homolog 5 (ALKBH5) are m^6^A RNA demethylases, which are involved in mammalian development, RNA metabolism and fertility (12,13). The human YTH-domain family 2 (YTHDF2) has recently been shown to specifically bind m^6^A-modified mRNA and to promote the decay of the bound mRNA (14,15). These discoveries present the first examples of reversible RNA modification and reveal the unique regulatory functions of reversible m^6^A methylation on mRNA and non-coding RNAs.

Transcriptome-wide profiling of m^6^A distributions in mammals and yeast further characterize the dynamic nature of m^6^A modification, which is enriched around the stop codon and at 3′ UTRs, as well as in long internal exons and at the transcription start site (11,15,16). m^6^A in *Arabidopsis thaliana* is also enriched around the stop codon, 3′ UTRs and around the start codon (17). The majority of m^6^A-peaks in these organisms harbor a consensus motif of RRACU (*R* = A/G).

Although m^6^A has been well documented in the rRNA in bacteria, its presence on mRNA is still elusive. In *Escherichia coli*, A1618 and A2030 of 23S rRNA are methylated by methyltransferases RlmF and RlmJ, respectively (18,19). Both deletion and overexpression of *rlmF* result in a loss of cell fitness and growth defect (18), while an *rlmJ* mutant shows mild phenotypes under various growth conditions (19). Interestingly, the modifications of m^2^A or m^8^A on A1607, A2503 and A2508 play important roles in antibiotic resistance, an extensively studied subject in microbiology during the last 10 years (20).

In order to investigate the potential presence and functions of m^6^A in bacterial mRNA, we calculated the m^6^A/A ratio in mRNA from seven diverse bacterial species, which reveal that m^6^A is an abundant mRNA modification in Gram-negative bacteria. High-resolution transcriptome-wide m^6^A profiling in two model bacteria *E. coli* and *Pseudomonas aeruginosa* reveal a conserved and distinct m^6^A distribution pattern. Most m^6^A-modified genes are involved in energy metabolism and small RNAs, suggesting potential functional roles of m^6^A in these processes.

## MATERIALS AND METHODS

### Bacterial strains and mRNA purification

The strains and culture conditions used in this study are listed in Table 1. Total RNA was purified from bacterial pellets of 2-ml culture by using an RNeasy Mini Kit (Qiagen) that removes tRNA. Two micrograms of total RNA were applied to a MICROExpressTM Bacterial mRNA Enrichment Kit (Life technologies). A Ribo-Zero™ rRNA Removal Kit (Bacteria) (Epicentre) was used in order to further remove remaining rRNA. All procedures in the manufacturer's protocols were strictly followed. In order to verify the removal of rRNA, a qPCR (7300 Real-Time PCR System, Applied Biosystems) was done against the rRNA background in order to check relative enrichment levels. One nanogram of either total RNA or purified mRNA from *E. coli* was used per qPCR reaction (Power SYBR Real-Time PCR mater mix, Life technologies). The primers used were 5′-CTCCTACGGGAGGCAGCAG-3′ and 5′-GTATTACCGCGGCGGCTG-3′. The *P. aeruginosa* MPAO1 strain were cultured overnight at different temperatures (37, 40, 42 or 45°C) and then subjected to the mRNA purification protocol described above in the temperature variation studies.

**Table 1.** Strains and growth conditions

Strain	Growth condition
*Escherichia coli* K-12 (CGSC)	LB, 37°C overnight
*E. coli* K-12 *rlmJ mutant* (CGSC)	LB, 37°C overnight
*E. coli* K-12 *rlmF mutant* (CGSC)	LB, 37°C overnight
*E. coli* K-12 *ksgA mutant* (CGSC)	LB, 37°C overnight
*E. coli* 5α	LB, 37°C overnight
*E. coli* XL-blue	LB, 37°C overnight
*Pseudomonas aeruginosa* MPAO1	LB, 37°C overnight
*P. aeruginosa* PA14	LB, 37°C overnight
*Pseudomonas syringae* pv. tomato DC3000	King's B medium, 28°C for 2 d
*Staphylococcus aureus* Newman	TSB medium, 37°C overnight
*S. aureus* USA100	TSB medium, 37°C overnight
*S. aureus* USA400	TSB medium, 37°C overnight
*S. aureus* USA700	TSB medium, 37°C overnight
*S. aureus* COL	TSB medium, 37°C overnight
*S. aureus* RN4220	TSB medium, 37°C overnight
*Bacillus subtilis*	LB, 37°C overnight
*Anabaena* sp. PCC 7120	Z8 medium, 25°C overnight
*Synechocystis* sp. PCC 6803	Z8 medium, 25°C overnight

### Ultra-high pressure liquid chromatography coupled with triple-quadrupole tandem mass spectrometry (UHPLC-QQQ-MS/MS) analysis for m^6^A/A ratio

The highly purified bacterial mRNA was subjected to an UHPLC-QQQ-MS/MS (Agilent) analysis. Two hundred ng of mRNA or rRNA (on the beads of the mRNA Enrichment Kit) were digested by nuclease P1 (2 U) in 40 μl of nuclease buffer (25 mM of NaCl and 2.5 mM of ZnCl_2_) at 37°C for 2 h, followed by the addition of NH_4_HCO_3_ (1 M, 2 μl) and alkaline phosphatase (0.5 U) at 37°C for 2 h. The nucleosides were separated by reverse phase ultra-performance liquid chromatography by a C18 column on an Agilent 6410 QQQ triple-quadrupole LC mass spectrometer in positive electrospray ionization mode. The nucleosides were quantified using the nucleoside-to-base ion mass transitions of 282 to 150 (m^6^A), 294 to 164 (m^6^_2_A) and 268 to 136 (A). Quantification was performed by comparison with the standard curve obtained from pure nucleoside standards. Three biological repeats have been performed for all bacterial strains.

### High-throughput and high-resolution m^6^A sequencing

Procedures were slightly modified from previously described protocols (21). In a 0.5-ml IP reaction, 5 μg purified bacterial mRNA and 15 μl of 0.5 mg/ml rabbit anti-m^6^A antibody (202003; Synaptic Systems) were incubated for 2 h at 4°C in IPP buffer (150 mM NaCl, 0.1% NP-40, 10 mM Tris–HCl, pH 7.4, 1 U/μl RNasin). The mixture was exposed to UV irradiation at 254 nm 3× (90 s each time), before RNase T1 (0.1 U/μl) digestion for 15 min at 22°C. After the digestion reaction was quenched on ice for 5 min, 200 μl pre-blocked protein A bead slurry was added into reaction for 1 h at 4°C. After washing thrice with IP wash buffer (50 mM HEPES-KOH, pH 7.5, 300 mM KCl, 0.05% NP-40, with proteinase inhibitor and RNasin), the beads were treated by a second round of RNase T1 digestion (15 U/μl) at 22°C for 15 min. The beads were cooled down on ice for 5 min and then thrice washed with high salt wash buffer (50 mM HEPES-KOH, pH 7.5, 500 mM KCl, 0.05% NP-40, with proteinase inhibitor and RNasin). The beads were then treated with Antarctic phosphatase (0.5 U/μl) for 20 min at 37°C. After dephosphorylation, beads were washed twice with phosphatase wash buffer (50 mM Tris–HCl, pH 7.5, 20 mM EGTA, 0.5% NP-40) and twice with PNK buffer without DTT (50 mM Tris–HCl, pH 7.5, 50 mM NaCl, 10 mM MgCl_2_). Polynucleotide kinase (1 U/μl) and 200 μM adenosine triphosphate was then added to the beads at 37°C for 15 min. The RNA fragments were further purified by proteinase K digestion and TRIzol extraction. For IP samples, small RNA libraries were made by using NEBNext^®^ Small RNA Library Prep Set for Illumina^®^ (NEB). The input samples followed the above procedures without anti-m^6^A antibody pull-down and RNase T1 digestion. Libraries for input samples were made by using TruSeq RNA Sample Preparation Kits (Illumina, non-strand specific). Six libraries were constructed, containing one control sample and two duplicate IP samples for each strain.

### Data analyses

All samples were sequenced using the HiSeq 2000 system (Illumina, with 50-bp and single end mode) at the Genomics Core Facility at the University of Chicago. FastQC was done to check the quality of each dataset; all datasets were obtained in high quality and can afford further reliable analyses. Two *E. coli* IP libraries obtained 1,718,364 mapped reads, and two *P. aeruginosa* libraries obtained 3,720,658 mapped reads. Sequence data were analyzed by following the procedures described previously (17). Briefly, Tophat (version 2.0.0, with the parameter: -p 8 –read-mismatches 2 –max-multihits 1) with Bowtie was run in order to align the input and IP-sequenced samples to the *E. coli* K-12 substr. MG1655 (ASM584v2, NC_000913.3) and *P. aeruginosa* PAO1 (ASM676v1, NC_002516.2) genomes and annotation files (22,23). In TopHat each read was only mapped to the genome once. The enriched peaks were identified using MACS software (version 2.0.0, with the parameter: callpeak -t ip.bam -c ck.bam -f BAM -g 6000000 –nomodel -n -p 1e-5) (24). Consensus sequence motifs were identified by using HOMER (version 4.7, with the parameter: -p 3 -rna -len 6) (25). A scrambled sequence was used as the background. Gene function analysis (GO enrichment) was performed with the online DAVID (version 6.7) tool (http://david.abcc.ncifcrf.gov/) (26). The m^6^A peaks were divided into three categories based on their relative positions in their corresponding genes: Overlap Start (±100 nucleotides around the start codon), Overlap End (±100 nucleotides around the stop codon) and Inside (other locations inside a coding region). The functional association with each gene was determined by NCBI annotation.

## RESULTS

### m^6^A is presented in mRNA of a wide range of bacterial species

Although m^6^A is the most abundant internal mRNA modification in eukaryotes, its potential presence in the kingdom of bacteria has yet to be investigated. To this end, we selected seven diverse model bacterial species (*E. coli, P. aeruginosa, Pseudomonas syringae, Staphylococcus aureus, Bacillus subtilis, Anabaena* sp. PCC 7120 and *Synechocystis* sp. PCC 6803) to grow in a common laboratory environment and measured their m^6^A/A ratios in purified mRNA. Unlike eukaryotes, bacterial mRNA lacks a poly(A) tail, which makes it challenging to purify mRNA. By following the protocols described in the Method section, we were able to remove >90% rRNA in the purified mRNA sample (Supplementary Figure S1).

We then tried to use an UHPLC-QQQ-MS/MS approach in order to quantify the m^6^A/A level in the bacterial mRNA samples that contain residual rRNA (<10%). Given that two m^6^A (catalyzed by RlmF and RlmJ) and two N^6^,N^6^-dimethladenosine (m^6^_2_A, catalyzed by KsgA) are known to be present in rRNA of *E. coli* and other related bacterial species (18,19,27), the values of m^6^_2_A levels can be used as an internal reference for the m^6^A level from the residue of rRNA presented in the purified mRNA. We first determined the m^6^_2_A/m^6^A ratio of rRNA as 1.30 in the wild-type strain and 2.04 in either an *rlmF* mutant or an *rlmJ* mutant. As a negative control, the m^6^_2_A modification was not detectable in a *ksgA* mutant (Supplementary Figure S2). Based on the m^6^_2_A/m^6^A ratio in rRNA, we were able to accurately calculate the real m^6^A/A level as ([m^6^A-m^6^_2_A/1.30]/A) in the purified mRNA samples.

**Figure 1.**
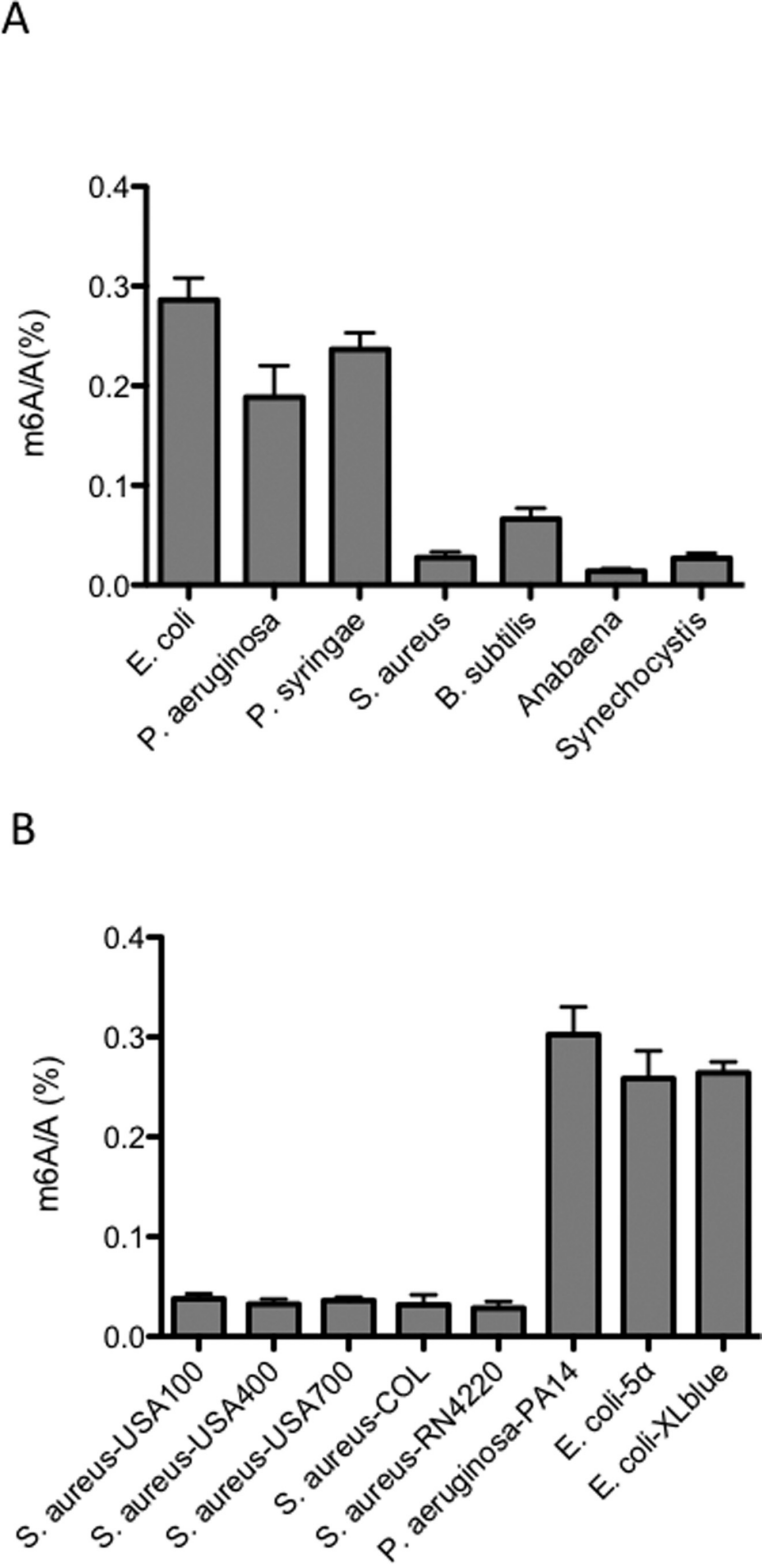
Presence of m^6^A in bacterial mRNA. (**A**) The m^6^A/A ratios of mRNA isolated from seven bacterial species. (**B**) The m^6^A/A ratios of mRNA isolated from different strains of *Escherichia coli* and *Pseudomonas aeruginosa*. Error bars represent standard deviations, which were calculated from three replicates.

We observed the presence of m^6^A in all of the tested bacterial species, whose m^6^A/A ratio varied within the range of 0.02–0.28% (Figure 1A). We obtained the m^6^A/A ratios in mRNA from three Gram-negative bacteria (*E. coli, P. aeruginosa* and *P. syringae*) (>0.2%) and from two Gram-positive bacteria (*S. aureus* and *B. subtilis*) (<0.08%). Unlike *E. coli* and *Pseudomonas* spp., two other Gram-negative cyanobacteria (*Anabaena* sp. PCC 7120 and *Synechocystis* sp. PCC 6803) showed low m^6^A/A ratios (<0.04%). In order to test if m^6^A/A ratios also vary among different strains in the same species, three strains of *E. coli* (K-12, 5α and XL-blue), two strains of *P. aeruginosa* (MPAO1 and PA14) and six strains of *S. aureus* (Newman, USA100, USA400, USA700, RN4220 and COL) were tested, all of which revealed a constant ratio in the same species (Figure 1B). These results clearly demonstrate the widespread occurrence of m^6^A in bacterial mRNA. Gram-negative bacteria tend to have higher m^6^A/A ratios in mRNA than Gram-positive bacteria. The high m^6^A/A ratio (>0.2%) in mRNA from *E. coli* and *Pseudomonas* spp. is comparable to that from eukaryotes (1), suggesting that m^6^A could be an important mRNA modification playing functional roles in these and other bacteria.

### m^6^A distribution exhibits a distinct topology in *E. coli*

To obtain the transcriptome-wide m^6^A map of *E. coli*, we employed an m^6^A-specific antibody for pull-down coupled with high-throughput sequencing (15,16). In order to obtain a high-resolution m^6^A-map, bacterial mRNAs were subjected to a modified photo-crosslinking-assisted m^6^A-seq approach (PA-m^6^A-seq), which significantly improves the m^6^A peak resolution from ∼200 nt to around 23 nt (21). In total, we identified 265 m^6^A peaks representing the transcripts of 213 genes in *E. coli* (Supplementary Table S1).

**Figure 2.**
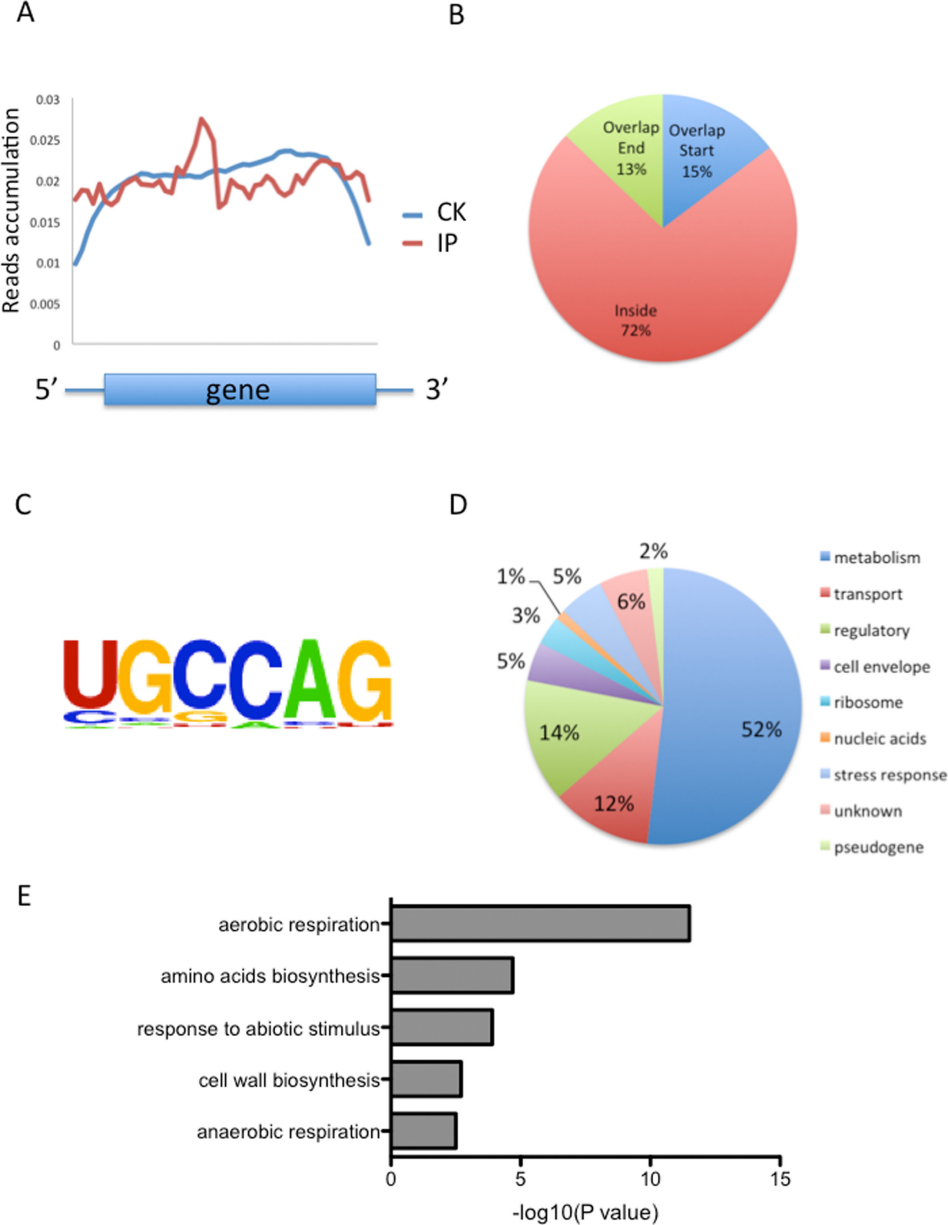
Overview of m^6^A methylome in *Escherichia coli*. (**A**) The m^6^A peak distribution within different gene contexts. The y-axis represents (number of reads/length unit)/(number of total reads), which is an indicator of the extent to which sequencing reads are enriched in different segments across the entire transcript. (**B**) Accumulation of m^6^A reads along transcripts. Each transcript is divided into three parts: Overlap Start, Inside and Overlap End. (**C**) The UGCCAG conserved sequence motif for m^6^A-containing peak regions. (**D**) Pie chart displaying the percentage of genes containing m^6^A peaks with functional categories. (**E**) GO-enrichment analysis of all the genes with m^6^A peaks. The effect size (number of enriched genes/total genes in the GO category) for each category is 15/45 (aerobic respiration), 15/122 (amino acids biosynthesis), 6/138 (response to abotic stimulus), 5/61 (cell wall biosynthesis) and 6/39 (anaerobic respiration), respectively. The statistical test (*P*-value) used by DAVID was the Fisher Exact test.

We next analyzed the distribution of m^6^A in the whole transcriptome of *E. coli*. We determined the distribution of m^6^A reads along transcripts in both the m^6^A-IP and non-IP (input) samples. Intriguingly, we found that reads from m^6^A-IP tend to be equally distributed throughout a gene, with a peak in the middle of open reading frames (ORFs) (Figure 2A). The prevalence is quite different from that observed in mammals, which accumulates around the stop codon and within 3′ UTRs (15,16). To further confirm the preferential locations of m^6^A on transcripts, we investigated the metagene profiles of m^6^A peaks. Consistent with the distribution of reads, m^6^A peaks are abundant inside ORF (72%), followed by regions at the start of gene (15%) and then the end of gene (13%) (Figure 2B).

We then used the HOMER tool to identify a leading m^6^A consensus sequence (UGCCAG, *P* < 1e-14), which could be found in more than 41.2% of all m^6^A peaks (Figure 2C and Supplementary Table S1) (25). This motif is different from the conserved one (RRACU, *R* = A/G) found in eukaryotes. The unique feature of the m^6^A distribution suggests a likely unique role of m^6^A in perhaps bacteria-specific pathways.

### m^6^A-containing mRNAs in important biological pathways in *E. coli*

**Figure 3.**
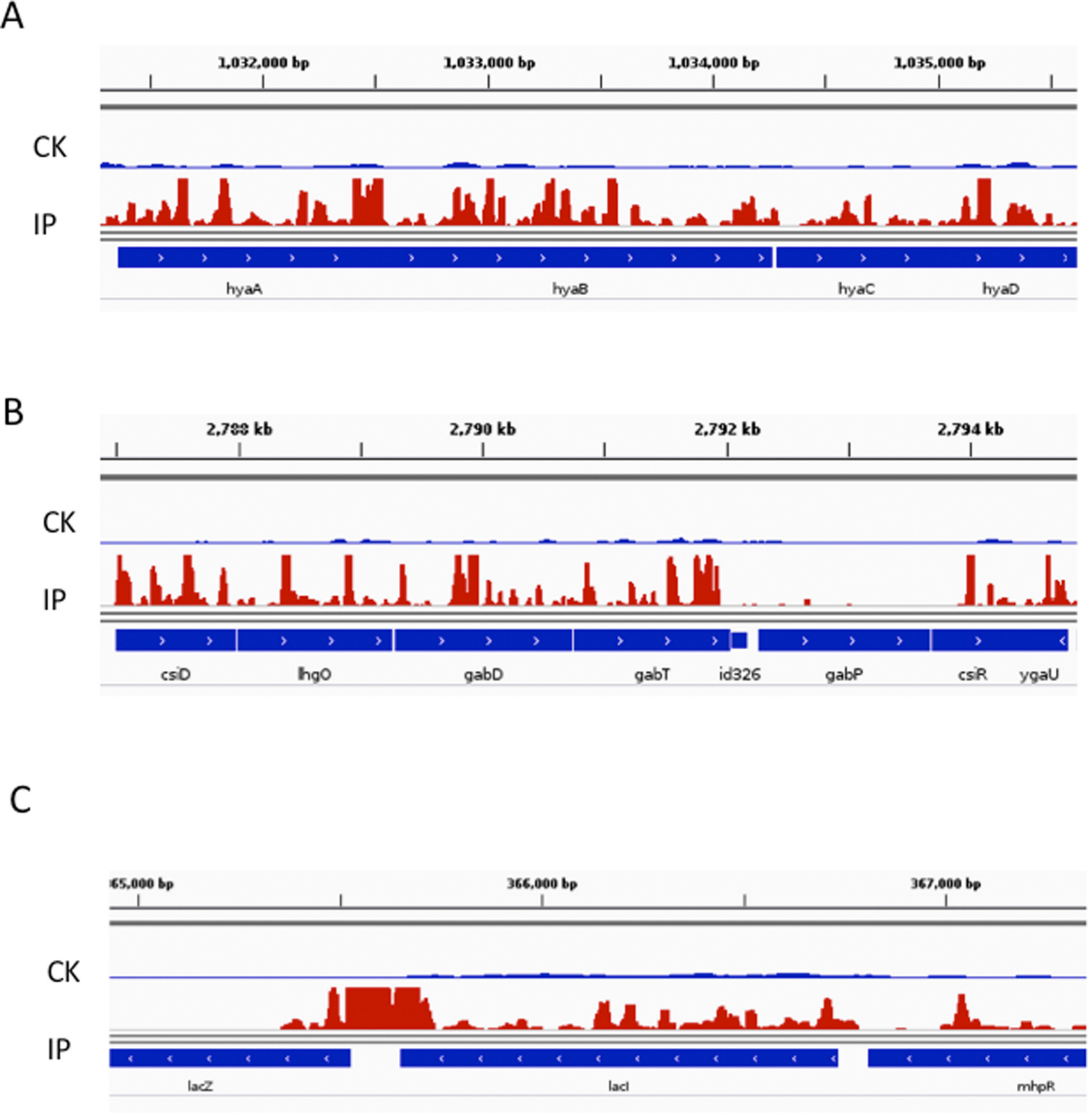
Accumulation of m^6^A reads in *hyaABCD* genes (**A**), *gabDT* (**B**) and *lacZI*(**C**) in *Escherichia coli* transcriptome. CK represents the control sample and IP represents ChIP-seq sample.

Diverse functions are encoded by m^6^A-containing genes, which include metabolism (52%), transportation (12%), gene regulation (11%), cell envelope (5%), ribosome (3%), nucleic acids (1%), stress response (5%), genes with unknown function/annotation (6%) and pseudogenes (2%) (Figure 2D). To further uncover potential functional insights on m^6^A in *E. coli*, we selected 213 m^6^A-containing transcripts and identified the enriched gene ontology (GO) terms using the DAVID tool (26). We found that these genes are highly enriched in aerobic respiration, amino acids biosynthesis, response to abiotic stresses, cell wall biosynthesis and anaerobic respiration (Figure 2E). The first two classes belong to housekeeping genes that are involved in central energy production and metabolism, while the latter three classes are bacteria-specific categories. The m^6^A distribution pattern suggests that m^6^A may play roles in these important biological pathways. For instance, hydrogenase 1 mediates hydrogen uptake and transport in the process of anaerobic respiration. Four (*hyaA, hyaB, hyaC* and *hyaD*) genes of the six-gene-operon encoding hydrogenase 1 contain multiple m^6^A peaks inside the transcript, suggesting a clustering of m^6^A in this operon (Figure 3A) (28). We also observed concentrated m^6^A peaks in *gabD* and *gabT* genes, which encode succinate-semialdehyde dehydrogenase and 4-aminobutyrate aminotransferase in the pathway of amino acid metabolism (Figure 3B) (29).

We next sought to determine if the unique m^6^A position patterns are related to bacteria-specific GO categories. As aforementioned, we classified genes into three subgroups according to the location of m^6^A peaks on a gene: Overlap Start (m^6^A peaks within 100-bp from the start codon), Overlap End (m^6^A peaks within 100-bp from the stop codon) and Inside (m^6^A peaks inside the coding region) (Figure 2B). We then performed GO-enrichment analysis for each subgroup. As expected, the same five GO categories were enriched in the Inside subgroup that consists of 72% of all m^6^A peaks. Two GO categories (aerobic respiration and stress responses) were enriched in the Overlap Start subgroup, while amino acids biosynthesis and response to stimulus were enriched in the Overlap End subgroup (Supplementary Figure S3).

Beside these five GO categories, we identified high m^6^A peaks in a group of functionally important genes, such as *lacZ* and *lacI* (Figure 3C). LacI negative regulates the classic lac operon (*lacZYA*) that is required for transport and metabolism of lactose (30). Interestingly, we also noticed 15 small RNAs carrying m^6^A modification (Supplementary Table S1). These newly found m^6^A marks in these transcripts could open a new angle to study novel regulatory roles in well-established pathways.

### Unique patterns of *P. aeruginosa* methylome

Given that *P. aeruginosa*, a widely-spread human opportunistic pathogen, also possesses a high m^6^A/A ratio in mRNA, we applied the same modified photo-crosslinking-assisted m^6^A-seq approach to obtain a high-resolution map of its m^6^A methylome. We identified 109 m^6^A peaks representing the transcripts of 68 genes in *P. aeruginosa* (Supplementary Table S2). The m^6^A-modified transcripts identified are around half of those in *E. coli*.

**Figure 4.**
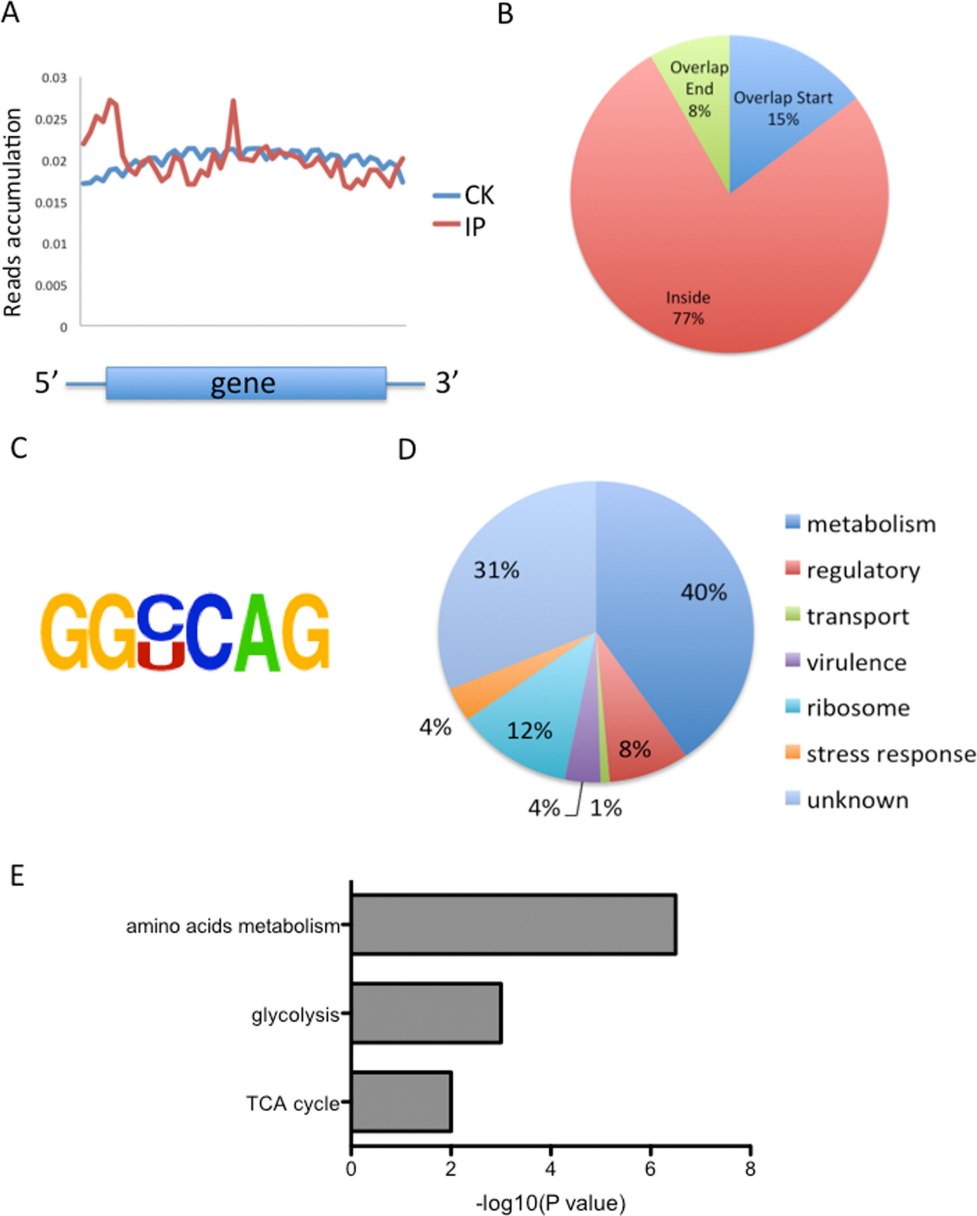
Overview of m^6^A methylome in *Pseudomonas aeruginosa*. (**A**) The m^6^A peak distribution within different gene contexts. The y-axis represents (number of reads/length unit)/(number of total reads), which is an indicator of the extent to which sequencing reads are enriched in different segments across the entire transcript. (**B**) Accumulation of m^6^A reads along transcripts. Each transcript is divided into three parts: Overlap Start, Inside and Overlap End. (**C**) The GGCCAG conserved sequence motif for m^6^A-containing peak regions. (**D**) Pie chart displaying the percentage of genes containing m^6^A peaks with functional categories. (**E**) KEGG-enrichment analysis of all the genes with m^6^A peaks. The effect size (number of enriched genes/total genes in the KEGG category) for each category is 7/148 (amino acids metabolism), 4/37 (glycolysis) and 3/56 (TCA cycle), respectively.

We next determined the distribution of m^6^A reads along transcripts in both the m^6^A-IP and non-IP (input) samples. Like in *E. coli*, we found that reads from m^6^A-IP are equally distributed throughout a gene, with two peaks in the middle of ORFs as well as in the beginning of genes (Figure 4A). m^6^A peaks are abundant inside ORF (77%), followed by the start of gene regions (15%) and end of gene regions (8%) (Figure 4B). We were also able to identify an m^6^A consensus sequence (GGYCAG, *Y* = C/U *P* < 1e-16), which were found in >70% of all m^6^A peaks (Figure 4C and Supplementary Table S2). This motif is almost identical to the one in *E. coli* (UGCCAG). A similar feature of the m^6^A distribution in both *E. coli* and *P. aeruginosa* indicates that m^6^A possesses functions unique to these bacteria.

**Figure 5.**
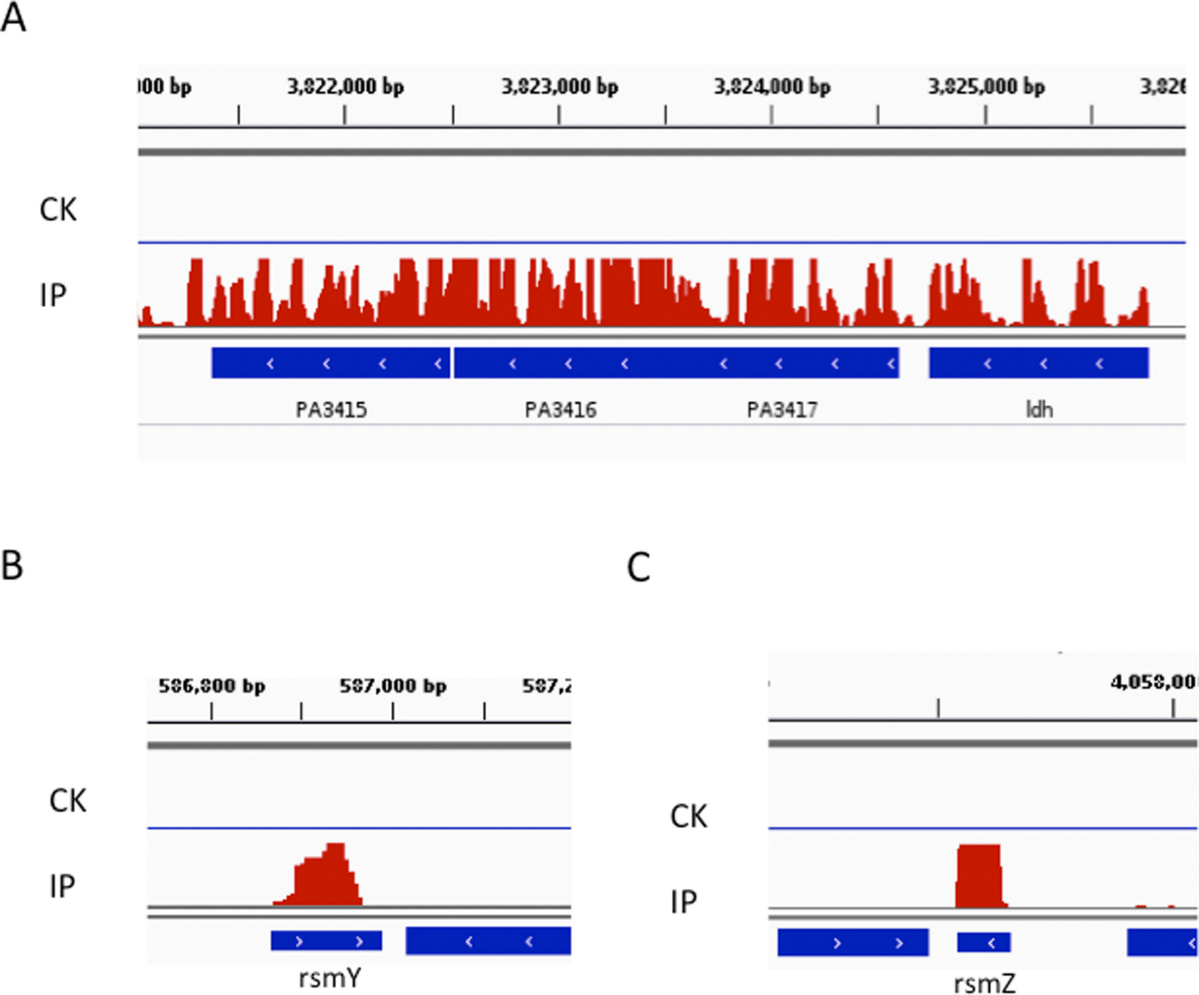
Accumulation of m^6^A reads in PA3415–3417 and *ldh* (**A**), *rsmY* (**B**) and *rsmZ* (**C**) in *Pseudomonas aeruginosa* transcriptome. CK represents control sample and IP represents ChIP-seq sample.

The m^6^A-containing genes cover different gene categories in *P. aeruginosa*, including metabolism (40%), gene regulation (8%), transportation (1%), virulence (4%), ribosome (12%), stress response (4%) and genes with unknown function/annotation (31%) (Figure 4D). DAVID analysis revealed significant GO enrichments in amino acids metabolism, glycolysis, and tricarboxylic acid (TCA) cycle (Figure 4E), all of which belong to housekeeping genes that are involved in central energy production and metabolism. The m^6^A pattern suggests that m^6^A may play roles in these essential pathways in *P. aeruginosa*. For instance, three adjacent genes (PA3415-PA3417 encoding branched-chain alpha-keto acid dehydrogenase, pyruvate dehydrogenase β and α subunit, respectively) involved in glycolysis and TCA carry numerous m^6^A peaks (Figure 5A) (31). We also observed multiple m^6^A peaks in the next downstream gene *ldh*, which encodes leucine dehydrogenase in the pathway of amino acid metabolism (Figure 5A). Beside these housekeeping genes, we identified high m^6^A peaks in two important small RNAs, namely RsmY and RsmZ, as well as in two key virulence genes *rhlA* and *rhlB* (Figure 5B).

### Temperature tunes m^6^A level in *P. aeruginosa*

**Figure 6.**
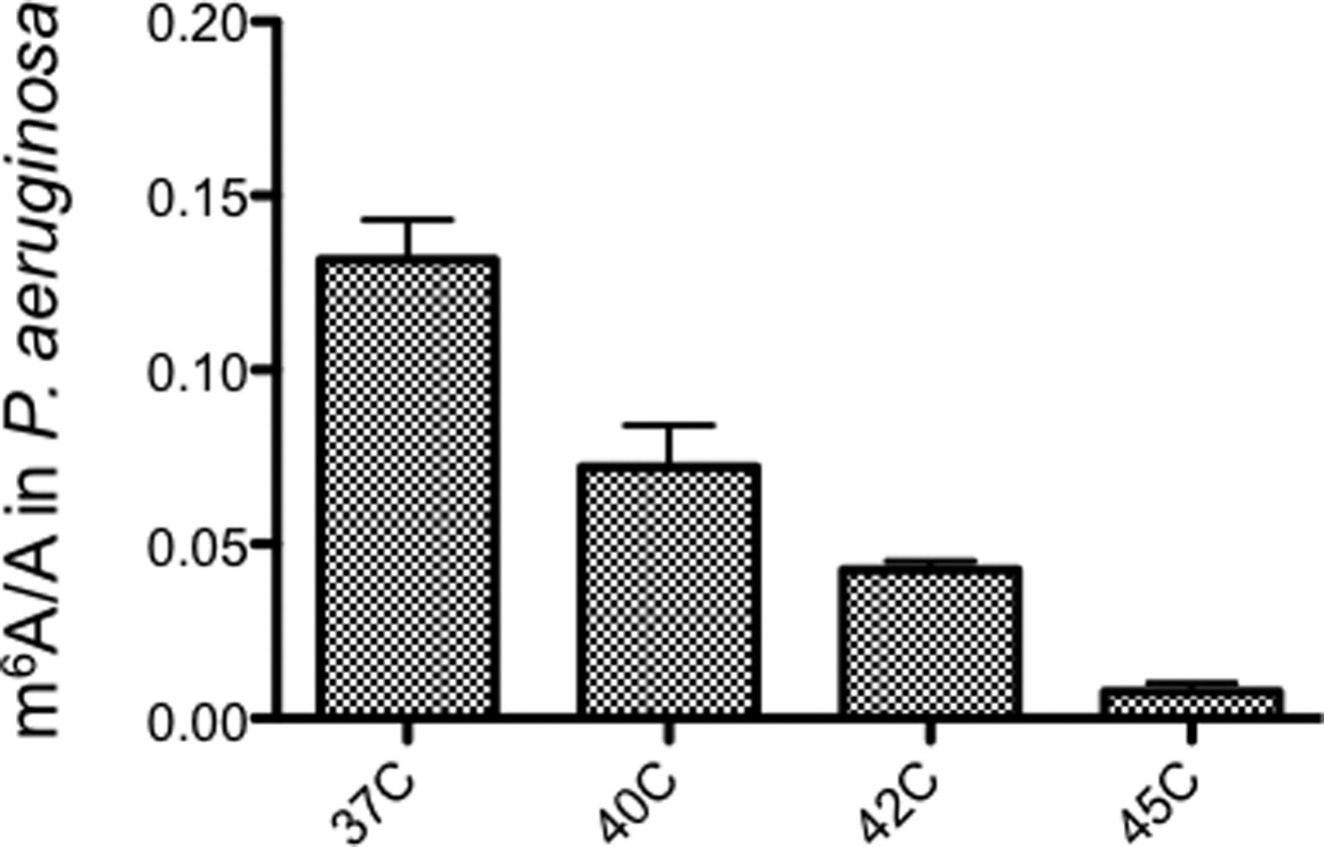
Growth temperature significantly affects the m^6^A/A ratio in *Pseudomonas aeruginosa*. Error bars are calculated from three replicates.

In humans and mice, dynamic changes of certain m^6^A peaks have been observed under dif­ferent stress conditions, indicating a link between m^6^A and stress response (15). In order to test if this trend also exists in bacteria, we measured the m^6^A/A ratios under a variety of growth environments or stress conditions (such as varying temperatures, different growth media, exposure to different antibiotics and oxidative stresses) for both *E. coli* and *P. aeruginosa*. For most tested conditions, we did not observe a significant difference in the m^6^A/A level compared to the normal condition (LB, mid-log phase, 37°C) for both bacteria. Interestingly, we noticed that increasing the culture temperature (from 37–45°C) led to a clear decrease in the m^6^A/A ratio in *P. aeruginosa* (Figure 6). Although *P. aeruginosa* still slowly grew, particularly at 45°C, the m^6^A modification was almost abolished.

## DISCUSSION

Recent discoveries and characterization of m^6^A erasers (demethylase), binders (m^6^A-specific binding protein) and writers (methyltransferase) as well as advances in profiling the m^6^A methylomes in eukaryotic systems reveal that m^6^A is a reversible and dynamic modification with important regulatory functions (1). On the other hand, the m^6^A methylomes in bacterial mRNA remain poorly studied. Here, we report the presence of m^6^A in a wide range of bacterial species and the ratio of m^6^A/A in mRNA from diverse bacterial strains vary within the range of 0.02–0.28%. We noticed that *S. aureus* and *B. subtilis* showed very low m^6^A/A ratios, which suggests that they may not possess an m^6^A methylase that could be present in the Gram-negative bacteria. Based on the genome annotation in NCBI, there are at least 43 proteins containing a S-adenosyl methionine (SAM)-binding domain in *E. coli* K-12, but only 24 in *S. aureus* USA300. We further present the high-resolution, transcriptome-wide m^6^A distributions in *E. coli* and human pathogen *P. aeruginosa*, which contain 265 and 109 peaks, respectively.

In order to provide additional insights into the overall m^6^A patterns in the bacterial kingdom, we compared these two newly identified methylomes. *E. coli* and *P. aeruginosa* share many similarities in their m^6^A distributions that are distinct from those of mammals: (i) a similar motif GCCAG instead of RRACU motif in mammals; (ii) most peaks are in the middle of coding region, while mammalian m^6^A peaks enrich around the stop codon and at 3′ UTRs; (iii) enrichment of GO categories of energy and amino acids metabolism; (iv) many small noncoding RNAs were found to carry m^6^A for both organisms. These shared characteristics suggest that other bacterial species, especially Gram-negative bacteria, may have similar m^6^A characteristics in mRNA. The new consensus sequence (GCCAG) is different from known rRNA methylation sites, including the two m^6^A sites on rRNA (CACA*GGU for RlmF and GUGA*AGA for RlmJ) and one on tRNA^val^ (UACA*AGG for YfiC). Given that our recent study demonstrates that m^6^A and its specific binding protein, YTHDF2, affect the translation status and lifetime of mRNA in eukaryotes (14), m^6^A may play a similar role in bacteria.

On the other hand, there are clear species-specific features of the m^6^A distribution between these two bacteria. Although the two species share major GO categories (energy and protein metabolism), we observed a very low rate of overlapping genes. Besides rRNA genes that are previously known to carry m^6^A modification, only one gene (*aceA* encoding isocitrate lyase) was shared between *E. coli* and *P. aeruginosa*. Each bacterium has distinct functional categories of mRNAs that carry m^6^A. For example, genes involved in cell wall biosynthesis and anaerobic respiration are enriched in the *E. coli* methylome only, while a group of virulence genes (RsmYZ and *rhlAB*) are enriched in the *P. aeruginosa* methylome (Figure 5B and C). RsmYZ binds to RsmA and dissociates RsmA away from its mRNA targets, which in turn tunes a group of important virulence pathways including Type III secretion system (T3SS) and biofilm formation (32,33). *rhlAB* encodes rhamnosyltransferase, producing rhamnolipid biosurfactants that are involved in uptake of hydrophobic substrates, virulence, biofilm and antibiotic resistance (34). m^6^A marks in these virulence genes could connect RNA modification to bacterial pathogenesis. Our result also suggests a relationship between m^6^A and *P. aeruginosa* adaption to temperature changes. Alternatively, the putative m^6^A methylase in *P. aeruginosa* may be inactive at high temperature.

Given that multiple known rRNA or tRNA adenine methylases have been characterized in *E. coli*, we measured the m^6^A/A ratios in two methylase mutants, *rlmF* and *rlmJ*. However, the ratios were not significantly lower than the wild-type strain, suggesting that they are not mRNA methyltransferases (Supplementary Figure S4). As a negative control, the *ksgA* mutant lost the m^6^_2_A modification and showed a higher m^6^A/A ratio than the other strains, suggesting a small content of rRNA in the mRNA sample. We also tried to look for bacterial homologs of mammalian m^6^A methyltransferases (METTL3, METTL14 and WTAP), but could not identify one, reminiscent of a distinct bacterial m^6^A motif that is different from that of mammals. These results suggest that bacterial m^6^A modification in mRNA is possessing of a mechanism that differs from eukaryotes.

Recent m^6^A profiling in yeast revealed dynamic changes in methylation during meiosis, which led us to test if bacterial m^6^A patterns also vary in different growth stage (11). To this end, we measured the m^6^A/A ratios during lag phase, log phase, stationary phase and death phase for both *E. coli* and *P. aeruginosa*. No significant difference was recorded throughout the bacterial growth curve, which indicates that m^6^A is a stable modification during bacterial growth.

The first bacterial m^6^A maps presented here provide a starting roadmap for uncovering bacterial distinct m^6^A functions in the future. Recent breakthroughs in the charac­terization of m^6^A-associated proteins as well as in the development of high-throughput assays in mammals present a very useful toolbox for us to study m^6^A in bacteria. Given the high abundance of m^6^A in numerous bacterial species, we foresee unique functions of m^6^A modification in mRNA in the wide bacteria kingdom.

## SUPPLEMENTARY DATA

Supplementary Data are available at NAR Online.

SUPPLEMENTARY DATA

## ACCESSION NUMBER

m^6^A-seq data are available in the National Center for Biotechnology Information Gene Expression Omnibus under series GSE65347.
