# Supplementary material for: Widespread occurrence of N6-methyladenosine in bacterial mRNA
Source: Nucleic Acids Res. 2015 Jun 11;43(13):6557–67. doi: 10.1093/nar/gkv596 (PMC4513869; doi:10.1093/nar/gkv596)
Supplement: SUPPLEMENTARY DATA [file supp_43_13_6557__index.html]

Widespread occurrence of N6-methyladenosine in bacterial mRNA — Widespread occurrence of N6-methyladenosine in bacterial mRNA — SUPPLEMENTARY DATA 

# Widespread occurrence of *N*6-methyladenosine in bacterial mRNA

## SUPPLEMENTARY DATA

- SUPPLEMENTARY DATA
- SUPPLEMENTARY DATA
