# Supplementary material for: Widespread occurrence of N6-methyladenosine in bacterial mRNA
Source: Nucleic Acids Res. 2015 Jun 11;43(13):6557–67. doi: 10.1093/nar/gkv596 (PMC4513869; doi:10.1093/nar/gkv596)
Supplement: SUPPLEMENTARY DATA [file supp_gkv596_nar-00630-v-2015-File009.docx]

**Widespread occurrence of *N*^6^-methyladenosine in bacterial mRNA**

Xin Deng^1,2#*^, Kai Chen^3,4#^, Guan-Zheng Luo^3,4^, Xiaocheng Weng^3,4^, Quanjiang Ji^3,4^, Tianhong Zhou^1,2^, Chuan He^3,4*^

**Figure S1.** qPCR verification of mRNA enrichment. qPCR was performed against the rRNA (primers targeting 16S rRNA) background to check the relative enrichment level of the mRNA sample from the wild type *E. coli*.

**Figure S2.** The ratios of m^6^_2_A/m^6^A in rRNA from the wild type, the *rlmJ* mutant and the *ksgA* mutant of *E. coli.*

**Figure S3.** GO-enrichment analysis of all *E. coli* genes with m^6^A peaks. (**A**) Overlap Start; (**B**) Overlap End; (**C**) Inside.


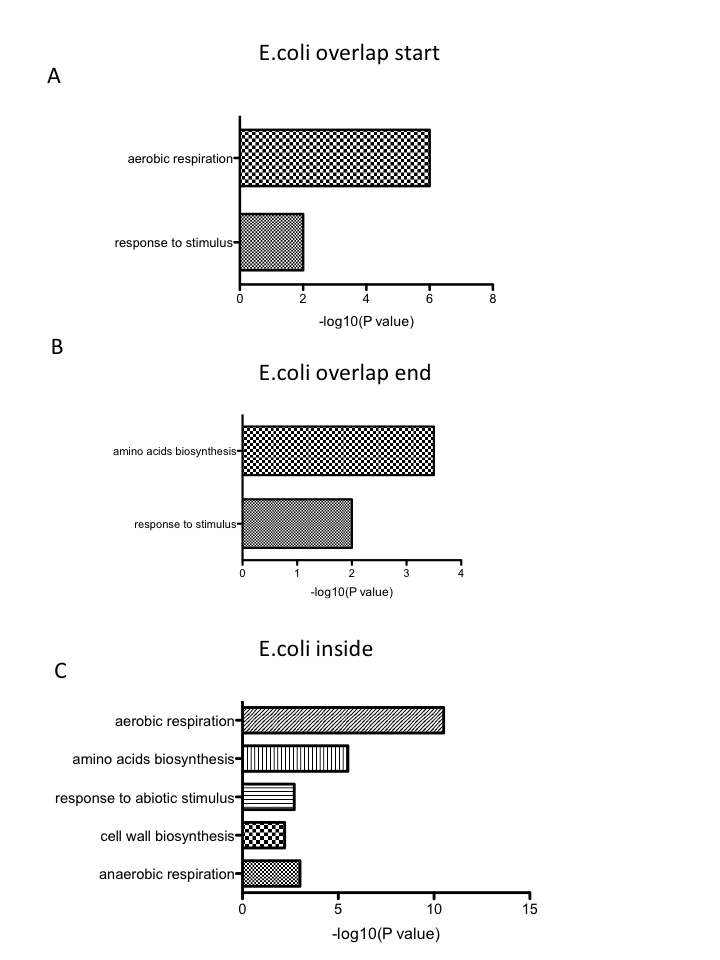


**Figure S4.** The m^6^A/A levels in mRNA from the wild type ([m^6^A-m^6^_2_A/1.30]/A), the *rlmF* mutant ([m^6^A-m^6^_2_A/2.04]/A), the *rlmJ* mutant ([m^6^A-m^6^_2_A/2.04]/A) and the *ksgA* mutant (m^6^A/A) of *E. coli.*

**Table S1.** Top m^6^A motifs (HOMER) in *E. coli.*

| Motif | % of targets | p-value | location |
| --- | --- | --- | --- |
| *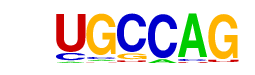* | 41.2% | 1e-14 | 68.1 +/- 79.4bp |
| *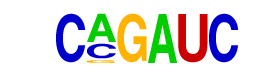* | 25.8% | 1e-19 | 41.4 +/- 47.1bp |

**Table S2.** Top m^6^A motifs (HOMER) in *P. aeruginosa.*

| Motif | % of targets | p-value | location |
| --- | --- | --- | --- |
| 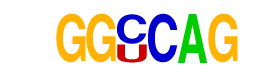 | 70.16% | 1e-16 | 702.7 +/- 823.5bp |
| 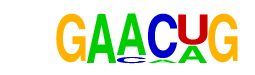 | 56.85% | 1e-30 | 759.0 +/- 884.9bp |
| 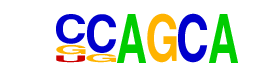 | 43.95% | 1e-13 | 795.2 +/- 910.8bp |
